# Chemical screening in Fabaceae identified GPM1 as a novel compound enhancing early graft adhesion

**DOI:** 10.1093/hr/uhag095

**Published:** 2026-03-13

**Authors:** Qianqian Luo, Xueyao Shu, Ayato Sato, Yaichi Kawakatsu, Kentaro Okada, Frank Opoku-Agyemang, Ken-ichi Kurotani, Michitaka Notaguchi

**Affiliations:** Graduate School of Bioagricultural Sciences, Nagoya University, Furo-cho, Chikusa-ku, Nagoya 464-8601, Japan; Graduate School of Bioagricultural Sciences, Nagoya University, Furo-cho, Chikusa-ku, Nagoya 464-8601, Japan; Institute of Transformative Bio-Molecules (WPI-ITbM), Nagoya University, Furo-cho, Chikusa-ku, Nagoya 464-8601, Japan; Center for One Medicine Innovative Translational Research (COMIT), Nagoya University, Furo-cho, Chikusa-ku, Nagoya 464-8601, Japan; Bioscience and Biotechnology Center, Nagoya University, Furo-cho, Chikusa-ku, Nagoya 464-8601, Japan; Bioscience and Biotechnology Center, Nagoya University, Furo-cho, Chikusa-ku, Nagoya 464-8601, Japan; Graduate School of Bioagricultural Sciences, Nagoya University, Furo-cho, Chikusa-ku, Nagoya 464-8601, Japan; Bioscience and Biotechnology Center, Nagoya University, Furo-cho, Chikusa-ku, Nagoya 464-8601, Japan; Center for Science Adventure and Collaborative Research Advancement, Graduate school of Science, Kyoto University, Kitashirakawa Oiwake-cho, Sakyo-ku, Kyoto 606-8502, Japan; Graduate School of Bioagricultural Sciences, Nagoya University, Furo-cho, Chikusa-ku, Nagoya 464-8601, Japan; Institute of Transformative Bio-Molecules (WPI-ITbM), Nagoya University, Furo-cho, Chikusa-ku, Nagoya 464-8601, Japan; Bioscience and Biotechnology Center, Nagoya University, Furo-cho, Chikusa-ku, Nagoya 464-8601, Japan; Department of Botany, Graduate School of Science, Kyoto University, Kitashirakawa Oiwake-cho, Sakyo-ku, Kyoto 606-8502, Japan; National Key Laboratory for Germplasm Innovation & Utilization of Horticultural Crops, College of Horticulture and Forestry Sciences, Huazhong Agricultural University, Wuhan, Hubei 430070, China

## Abstract

Plant grafting is a horticultural technique used to join different plants with desirable traits. However, graft incompatibility limits its application, especially in agriculturally important Fabaceae species. To enhance grafting efficiency, we conducted chemical screening utilizing an *in vitro* grafting (IVG) system in Fabaceae. In this study, we screened 3000 artificial chemical compounds and identified a compound, designated graft-promoting molecule 1 (GPM1), which enhanced graft adhesion in Fabaceae species—including *Phaseolus coccineus*, *Vigna unguiculata*, *Vigna angularis*, and *Glycine max*—as well as in *V. unguiculata*/*G. max* hetero-IVGs at 5 days after grafting (DAG). Notably, GPM1 also increased adhesive force in *Nicotiana benthamiana* IVGs and improved survival rates in *Arabidopsis thaliana* micrografting, indicating that its activity is not restricted to Fabaceae. Transcriptome analysis of *P. coccineus* IVGs at 1 DAG revealed that application of GPM1 induced the upregulation of cell wall modification genes, including *PvEXPA5*, *PvEXPA22,* and *PvEXPA25*. In contrast, treatment with 2,4-dichlorophenoxyacetic acid (2,4-D) induced a broader transcriptional response, predominantly upregulating genes related to cell division. In *G. max* stem grafting, GPM1 enhanced scion growth and promoted the formation of larger callus cells at the graft junction. Moreover, qRT-PCR analysis revealed that GPM1 significantly upregulated *Glyma.07G229000*, a homolog of *PvEXPA5*. The upregulation of cell wall-associated genes by GPM1 is consistent with a role in early graft union formation, potentially by facilitating tissue adhesion at the graft interface. Collectively, this study identifies GPM1 as a chemical regulator that enhances graft adhesion and provides insight into molecular processes associated with early graft adhesion.

## Introduction

Grafting is a widely employed horticultural technique used to join distinct plant parts to form a single chimeric organism with desirable traits. These traits include biotic and abiotic stress resistance [1, 2] and high yield and improved quality through grafting [3, 4]. Successful graft establishment mainly depends on efficient tissue adhesion, callus formation, and vascular reconnection between the scion and rootstock [5, 6]. Consequently, the successful union formation affects major physiological processes, such as water uptake, nutrient mobilization, and root-shoot signaling, which eventually results in scion growth and/or fruit setting [7–9]. Grafting is well established in woody plants as well as herbaceous plant families, such as Solanaceae, Cucurbitaceae, and Rosaceae [10]. Its successful adoption has resulted in a substantial increase in the application of grafting in commercial vegetable production over the last three decades, thereby driving extensive research efforts in the field [10, 11]. However, the application of grafting in the Fabaceae family remains limited [12].

Fabaceae (Leguminosae) is the third largest angiosperm family, with an estimated 19 000 species, including agriculturally important crops, such as soybean (*Glycine max*), common bean (*Phaseolus vulgaris*), runner bean (*Phaseolus coccineus*), pea (*Pisum sativum*), lentil (*Lens culinaris*), chickpea (*Cicer arietinum*), and alfalfa (*Medicago sativa*) [13, 14]. Representing ~8% of global vascular plants, Fabaceae exhibit extensive morphological, genetic, physiological, and ecological diversity [15]. These plants contribute to sustainable agriculture through symbiotic nitrogen fixation with *Rhizobium* and *Bradyrhizobium* species [16, 17] and are vital sources of proteins, calories, vitamins, minerals, and fibers, supporting global food security [18].

Grafting in legumes has been used as a research tool to investigate shoot-root interactions involved in nodulation and development, particularly in model species such as *Lotus japonicus* [19], *Medicago truncatula* [20], and *G. max* [21]. It has gained attention for practical agricultural applications, including seed multiplication and the maintenance of interspecific hybrids in *P. vulgaris* [22], and mitigating soil degradation, salinity, and soilborne diseases by using *P. coccineus* as rootstock [23]. However, interspecies grafting study within legumes has shown that graft compatibility and vascular reconnection vary markedly even among closely related species, which constrained extensive practical application of grafting in Fabaceae [24]. During graft union formation, inadequate tissue adhesion, an early grafting step, impairs callus formation and functional vascular development, resulting in unsuccessful grafting. In a wide range of interfamily graft practices using *Nicotiana* species, some of Fabaceae plants, e.g. *G. max* and *Lotus japonicus*, have been identified to exhibit strong incompatible cell–cell adhesion resulting from necrotic layer formation at the graft boundary coupled with folded cell wall remnants caused by graft injury compared with other plant families [25]. Xylem bridge connection observations revealed fewer xylem cells differentiated from Fabaceae scions, resulting in the absence of xylem bridge formation and secondary xylem development, indicating the failure to establish continuous vascular connections [9]. Therefore, there is a need to elucidate the molecular and physiological mechanisms that govern graft compatibility and to develop strategies to enhance grafting efficiency across Fabaceae species.

Successful graft union formation requires coordination of several biological processes (BPs), including wound healing, cell wall remodeling, callus formation, tissue adhesion, and vascular development [26, 27]. This adhesion begins at the cellular level with cell–cell adhesion mediated by cell wall remodeling, involving expansins (EXPA), xyloglucan endotransglucosylase/hydrolases (XTHs), pectin methylesterases (PMEs), and β-1,4-glucanases, which together promote tissue adhesion and reorganization at the graft interface [25, 28, 29]. Also, the accumulation of auxins at the graft junction has been identified to stimulates cell division, vascular strand formation, and tissue patterning [30, 31]. Efficient tissue adhesion enables subsequent crucial processes, such as vascular tissue differentiation and long-distance nutrient and signal transport. Exogenous application of plant hormones and enzymatic treatments have been utilized to enhance tissue adhesion and improve grafting success [32]. Specifically, application of auxins, such as 2,4-dichlorophenoxyacetic acid (2,4-D) and cellulase, positively influenced graft union adhesion with an additive effect when applied together [32]. However, there is no evidence of exogenous phytohormones, enzyme agents, and chemical compounds application in Fabaceae grafting, and their roles in Fabaceae remain underexplored. In this study, by employing a chemical biology approach, we aimed to identify novel chemical compounds that can enhance tissue adhesion in Fabaceae grafting using an *in vitro* grafting (IVG) system.

Given the complex interplay between hormonal signaling and cell wall dynamics during graft formation, chemical biology approaches using IVG offer a promising strategy to identify molecules capable of enhancing tissue adhesion during graft formation in Fabaceae. Chemical biology, in which researchers use small compounds to understand BPs, has expanded in recent years [33–35]. Plant researchers have identified compounds that mimic plant hormones [36–40]. To our knowledge, compounds regulating tissue adhesion during Fabaceae grafting have not yet been identified. The IVG technique involves grafting a microscion onto a microrootstock under sterile conditions [41]. Originally developed in citrus for virus elimination [42], IVG has been applied to various species to detect graft incompatibility using markers, such as phenolic accumulation, delayed cambium formation, and reduced plasmodesmal connectivity [43–45]. The modified IVG system in *Nicotiana benthamiana* allows quantitative evaluation of graft adhesion (defined in this study as the physical attachment between scion and rootstock) within 7 days using a force gauge [32], providing a high-throughput, robust, and reproducible platform for screening chemical compounds that enhance tissue adhesion.

In this study, we established a chemical screening method using a modified IVG system optimized for Fabaceae. We utilized a ITbM (The Institute of Transformative Bio-Molecules) chemical library consisting of 3000 structurally diverse synthetic small molecules with molecular weights of 500 or less, to identify those that promote tissue adhesion. We identified a novel compound designated as graft-promoting molecule 1 (GPM1), which significantly enhanced tissue adhesion in *P. coccineus* grafts. GPM1 efficacy was further confirmed across multiple Fabaceae species, including *Vigna unguiculata*, *Vigna angularis*, and *G. max*, as well as in *V. unguiculata*/*G. max* hetero-IVGs. In addition, graft adhesion was assessed in *N. benthamiana* IVGs and survival rate was examined in *Arabidopsis thaliana* micrografting. Transcriptomic and qRT-PCR analysis at early stages of grafting revealed upregulation of cell wall modification genes, including several expansin genes. Finally, we showed that GPM1 treatment enhanced scion growth and callus formation at the graft junction in *G. max*. These findings introduce GPM1 as a novel tissue adhesion-enhancing compound associated with the regulation of cell expansion process during grafting and suggest a potential strategy to improve grafting efficiency in Fabaceae crops.

## Results

### Establishment of chemical screening method

**Figure 1:**
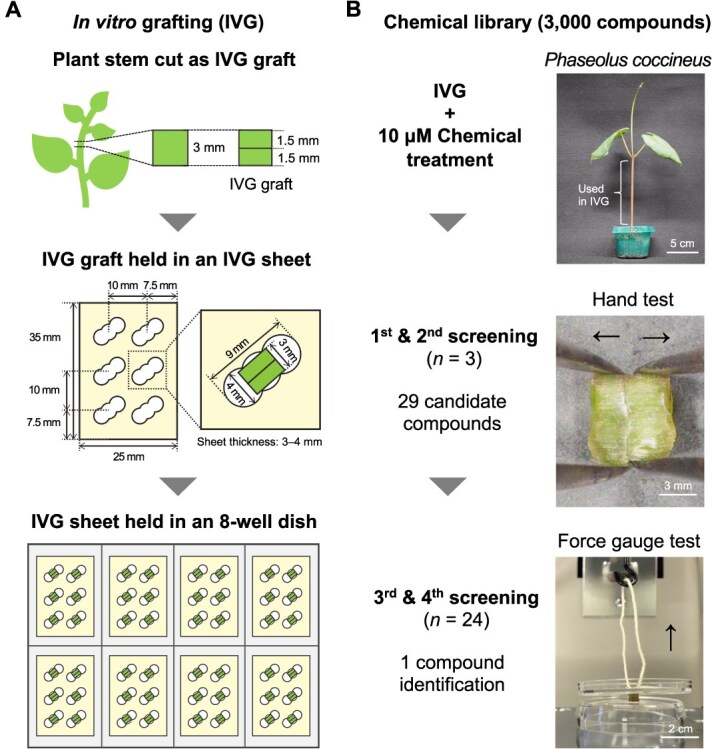
Chemical screening for graft-promoting molecules using an IVG system. (A) IVG method used in this study. Plant stems were cut into 3 mm segments, halved horizontally, and reassembled to form IVG grafts, which were placed into six cutouts on an IVG sheet within an 8-well dish containing culture medium with test compounds. (B) Screening workflow to identify graft-promoting molecules. Total 3000 compounds from the ITbM library were tested at 10 μM concentration using 10-day-old *P. coccineus* epicotyls. Grafts were incubated at 27°C under continuous light for 5 days. In the first and second screenings, each compound was tested in 3 IVG grafts. Manual assessment of graft adhesion was performed using tweezers to gently pull the graft apart; compounds that adhered in at least two grafts were selected as candidates. The 29 selected compounds proceeded to the third and fourth screenings, with 24 IVG grafts per compound. Adhesive force was quantified using a force gauge. One novel compound was ultimately identified as a graft-promoting molecule. Scale bars: 5 cm, 3 mm, 2 cm.

To screen for compounds that enhance graft tissue adesion, we established a robust chemical screening method by employing an IVG system [32]. For the IVG system, a custom-designed IVG sheet (25 × 35 mm), featuring six cutouts to hold grafted samples, was placed in an 8-well dish, allowing simultaneous preparation of up to 48 IVG grafts with eight different compounds (Fig. 1A). This setup enabled high-throughput chemical screening to identify graft-promoting molecules. For plant material, we selected *P. coccineus* due to its favorable growth characteristics for IVG. The plants reach ~20 cm in height within 10 days after sowing, produce few lateral branches, and have consistently thick stems, with the epicotyl measuring around 0.5 cm in diameter. These features make *P. coccineus* particularly suitable for consistent and reproducible chemical screening (Fig. 1B).

To determine the optimal time point for measuring adhesive force in *P. coccineus* IVG grafts, we conducted a time-course analysis for both mock and 0.5 μM 2,4-D treated samples. Using a force gauge, adhesive force was measured from 1 to 7 days after grafting (DAG). While mock-treated grafts showed gradual increases of adhesive force (~0.05 N at 5 DAG), 2,4-D-treated grafts displayed significantly higher adhesive force from 3 DAG and later (~0.1 N at 5 DAG) (Fig. S1, Movies S1 and S2). From these results, we selected 5 DAG as the optimal time point for measuring adhesive force in subsequent chemical screening experiments.

### Chemical screening identified GPM1

To identify compounds that enhance graft tissue adhesion, we screened 3000 artificially synthesized chemicals at 10 μM using mock (0.1% DMSO) and 0.5 μM 2,4-D as negative and positive controls, respectively. *P. coccineus* IVG samples were incubated in compound-containing media in 8-well dishes for 5 days at 27°C under continuous light. Adhesion in the first and second screening was assessed qualitatively using a ‘hand test’, where grafts were gently pulled apart with tweezers. Three IVG grafts per compound were prepared, and compounds were considered candidates if at least two of three grafts showed strong adhesion, resulting in 29 candidates for further testing.

**Figure 2:**
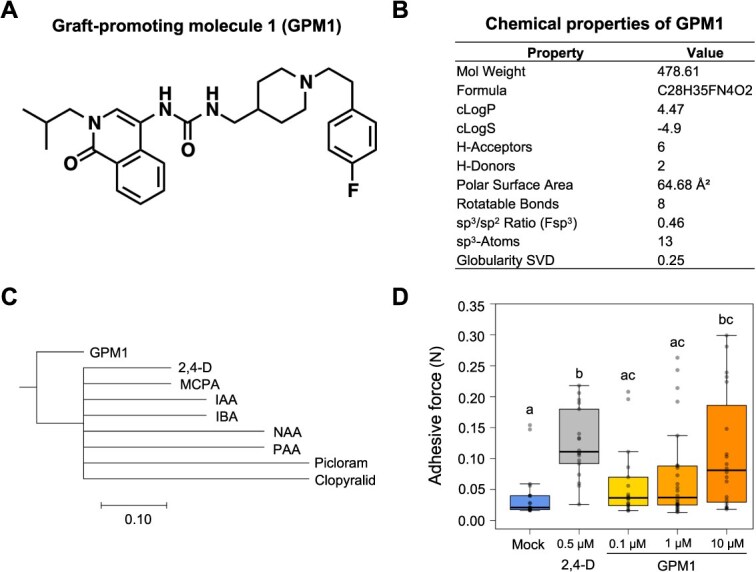
Identification and dose response analysis of a graft-promoting molecule (GPM1). (A) Chemical structure of the identified graft-promoting molecule, GPM1. (B) Chemical properties of GPM1. (C) Hierarchical clustering of GPM1 and auxins based on chemical structure similarity (Tanimoto score). (D) Dose–response effect of GPM1 on graft adhesion in the IVG system using epicotyls of 10-day-old *P. coccineus.* Adhesive force was measured at 5 DAG. Treatments included mock (0.1% DMSO), 0.5 μM 2,4-D, and GPM1 at 0.1, 1, and 10 μM. *n* = 18–26. Different letters indicate statistically significant differences (Steel-Dwass test, *P* < 0.05).

To identify the superior chemical compounds from the 29 candidates, a total of 24 IVG grafts were prepared for each compound for the third and fourth screening rounds. The adhesive force of *P. coccineus* IVG stem tissues were quantitatively measured using a force gauge. For the measurement, both sides of the IVG stem tissues were firmly fixed to the surface of plastic Petri dishes using instant adhesive, with a string attached to the dish lid hooked to the force gauge. The force gauge pulled the string upward at a constant speed (5 mm·s^−1^) and the absolute difference between the initial and final force values were defined as the adhesive force of each treated IVG sample. At 5 DAG, one compound, designated graft-promoting molecule 1 (GPM1, N-[1,2-dihydro-2-(2-methylpropyl)-1-oxo-4-isoquinolinyl]-N′-[[1-[2-(4-fluorophenyl)ethyl]-4-piperidinyl]methyl]-urea, Chemical Abstracts Service Registry Number (CAS RN): 894581-47-6), significantly enhanced graft adhesion (Figs 1B and 2A).

GPM1 has a molecular weight of 478.61, moderate lipophilicity (cLogP = 4.47), a polar surface area (PSA) of 64.68 Å^2^, eight rotatable bonds, and an Fsp^3^ (fraction of sp^3^-hybridized carbons) value of 0.46 (Fig. 2B). Hierarchical clustering analysis on the chemical structure revealed that GPM1 is structurally distinct from known auxins, including natural auxins (IAA, IBA, PAA) and synthetic analogs (2,4-D, MCPA, NAA, picloram, and clopyralid) (Fig. 2C). Dose–response analysis further demonstrated that GPM1 significantly increased adhesive force at a concentration of 10 μM (Fig. 2D). To assess potential auxin-like activity of GPM1, *A. thaliana* seedlings were treated with 2,4-D or GPM1 at concentrations including 0.01, 0.1, 1, and 10 μM. Consistent with known auxin activity, 2,4-D significantly induced concentration-dependent inhibition of primary root elongation at 0.1 μM and higher. However, GPM1 had no significant effect on primary root length at any tested concentration compared with the mock (Fig. S2A and B). To assess auxin-responsive signaling, a *DR5::GFP* reporter line was examined after treatment with 2,4-D or GPM1 at 0.1, 1, and 10 μM. As expected, 2,4-D strongly induced the GFP fluorescence signal across root tissues. In contrast, GPM1 did not cause detectable expansion of the GFP fluorescence signal at any concentration tested (Fig. S2C). These results suggest that GPM1 does not activate canonical auxin-responsive pathways, highlighting its unique functional activity and greater physiological selectivity.

### GPM1 promoted graft adhesion in multiple species

**Figure 3:**
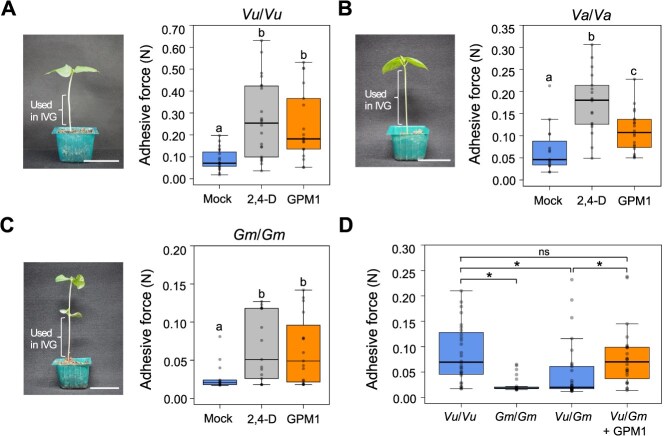
GPM1 enhanced graft adhesion in multiple Fabaceae species at 5 DAG. (A) IVG of *Vigna unguiculata* (*Vu*) using hypocotyls of 10-day-old seedlings (*n* = 18–22). (B) IVG of *Vigna angularis* (*Va*) using 20-day-old hypocotyls (*n* = 15–20). (C) IVG of *Glycine max* (*Gm*) using 10-day-old hypocotyls (*n* = 14–19). Representative seedling images are shown in A–C. Scale bars = 5 cm. Treatments included mock (0.1% DMSO), 0.5 μM 2,4-D, and 10 μM GPM1. Different letters indicate statistically significant differences between treatments (Steel-Dwass’ test, *P* < 0.05). (D) IVG of homografts and heterografts between *Vu* and *Gm*, with *Vu* as the scion in heterografts (*n* = 23–29). Treatments included mock and 10 μM GPM1. ns, not significant. Asterisks indicate significant differences (Mann–Whitney *U* test, *P* < 0.05).

To assess the broader efficacy of GPM1 in promoting graft tissue adhesion in Fabacae species, we evaluated its effects in three additional species of agricultural importance including *V. unguiculata*, *V. angularis*, and *G. max*. The IVG stems from each species were treated with mock (0.1% DMSO), 0.5 μM 2,4-D, or 10 μM GPM1, and graft tissue adhesion was quantified by measuring adhesive force at 5 DAG. Both 2,4-D and GPM1 significantly increased graft tissue adhesion compared to the mock treatment across all three species (Fig. 3A–C). These results demonstrate that GPM1 has a broad applicability within the Fabaceae family in promoting graft tissue adhesion.

To further investigate the activity of GPM1 in heterograft tissue adhesion, we prepared IVG stem homografts of *V. unguiculata* and *G. max* and heterografts of *V. unguiculata* scion and *G. max* rootstock. The species *V. unguiculata* and *G. max* were selected because their tissue adhesion measurements were contrasting. *V. unguiculata* exhibited the high adhesion response (0.18 N), while *G. max* showed the low (0.04 N) (Fig. 3A and C). Since cell proliferation and tissue adhesion are mostly relied on the action in the scion side in grafting, *V. unguiculata* was selected as the scion in heterografting. Although the stem sizes of these two species were not significantly different (Fig. 3A and C), the adhesive force of *V. unguiculata*/*G. max* heterografts was markedly lower than that of *V. unguiculata* homografts and was comparable to that observed in *G. max* homografts (Fig. 3D). However, the *V. unguiculata*/*G. max* IVGs treated with 10 μM GPM1 showed significantly higher adhesive force than the mock-treated hetero-IVGs (Fig. 3D), indicating that GPM1 can enhance tissue adhesion even in heterograft which showed low adhesion capacity.

To test whether the graft-promoting effect of GPM1 extends beyond Fabaceae, we examined its activity in *N. benthamiana* and *A. thaliana*. In *N. benthamiana* IVGs, GPM1 increased adhesive force at 7 DAG compared with the mock (Fig. S3). In addition, GPM1 led to a higher survival rate in *A. thaliana* micrografts at 14 DAG (Fig. S4). These results suggest that the graft-promoting effect of GPM1 is observed across phylogenetically distinct species.

### GPM1 induced cell wall-related genes in RNA-seq analysis

**Figure 4:**
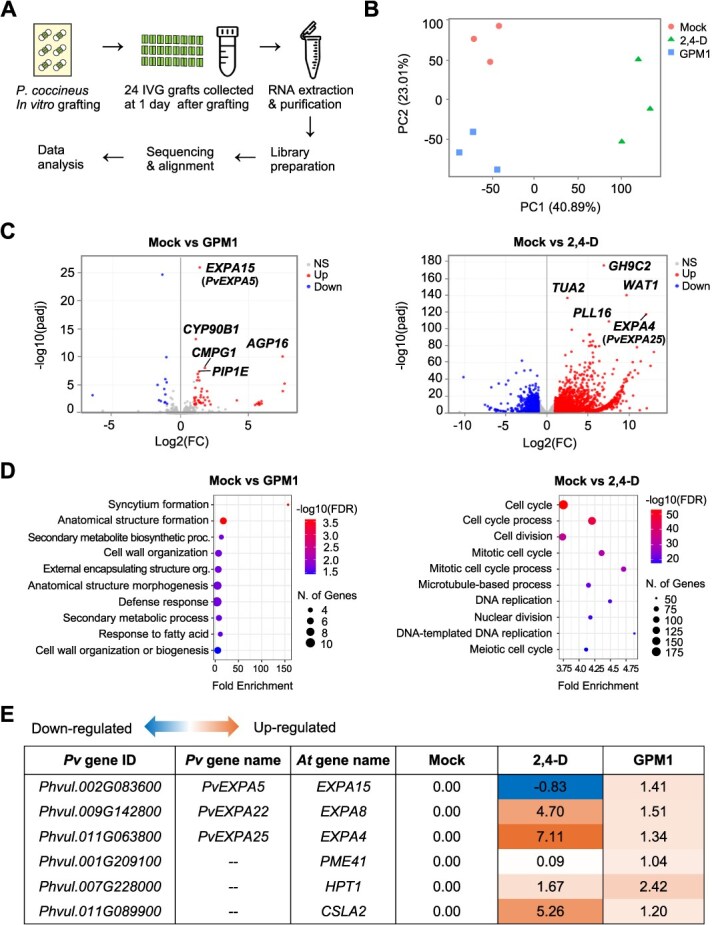
Transcriptomic profiling of IVG grafts treated with GPM1 and 2,4-D in *P*. *coccineus*. (A) RNA-seq workflow. (B) Principal component analysis of gene expression in IVG grafts treated with mock (0.1% DMSO), 0.5 μM 2,4-D, or 10 μM GPM1 at 1 DAG. (C) Volcano plots of DEGs in GPM1 and 2,4-D treated samples relative to the mock samples. Red and blue dots indicate significantly up- and downregulated genes (adjusted *P* < 0.05, |log_2_ FC| > 1); grey dots represent non-significant. Top five upregulated genes are labeled using *A. thaliana* annotation names. (D) Gene Ontology enrichment analysis of upregulated genes. Top 10 Biological Process terms are shown for GPM1 and 2,4-D. (E) Heatmap showing the expression of genes in the ‘cell wall organization’ category under mock, 2,4-D, and GPM1 treatments. *P. vulgaris* gene nomenclature follows the Expansin Gene Family Database. Colors represent log_2_ FC in transcript levels at 1 DAG relative to the mock samples.

To investigate the molecular mechanisms underlying GPM1-induced promotion of graft tissue adhesion, we performed transcriptome analysis of *P. coccineus* IVG stems at 1 DAG under three treatments: mock (0.1% DMSO), 0.5 μM 2,4-D, and 10 μM GPM1. For each treatment, three biological replicates were prepared, and RNA-seq libraries were sequenced to a depth of ~10 million reads per sample. RNA was mapped to the *P. vulgaris* reference genome (Pvulgaris_442_v2.1) (Fig. 4A). Principal component (PC) analysis showed clear separation among treatments, with PC1 and PC2 explaining over 63% of the variance. Mock samples (red circles) clustered at the upper left, 2,4-D-treated samples (green triangles) were positioned toward the right along PC1, and GPM1-treated samples (blue squares) formed a separate cluster at the lower left along PC2. This pattern indicates that GPM1 triggers a transcriptional response that is distinguishable from both mock and 2,4-D treatments (Fig. 4B).

The differentially expressed genes (DEGs) specifically regulated by the treatments during tissue adhesion were identified by comparing each treatment to the mock. In GPM1-treated samples, a total of 64 DEGs were identified with 49 genes significantly upregulated and 15 downregulated genes relative to mock (adjusted *P* < 0.05, |log_2_ fold change (FC)| > 1) (Fig. 4C). In contrast, 2,4-D induced a much broader transcriptional response with a total of 3262 DEGs including 2404 upregulated genes and 858 downregulated genes compared to mock (Fig. 4C, Tables S1–S4). To gain functional insights into these transcriptional changes, gene ontology (GO) enrichment analysis was performed and the BP category is presented. Among the GPM1-induced upregulated genes, the top five significantly enriched BPs were ‘syncytium formation’, ‘anatomical structure formation’, ‘secondary metabolite biosynthetic process’, ‘cell wall organization’, and ‘external encapsulating structure organization’ (Fig. 4D). These significantly enriched BPs revealed that GPM1 upregulated genes associated with cell wall modification, including several expansin genes, *Phvul.002G083600* (*PvEXPA5*), *Phvul.009G142800* (*PvEXPA22*), and *Phvul.011G063800* (*PvEXPA25*), a pectin methylesterase gene, *Phvul.001G209100* (a homolog of *A. thaliana PME41*), a homogentisate phytyltransferase gene, *Phvul.007G228000* (a homolog of *A. thaliana HPT1*), and a cellulose synthase-like gene, *Phvul.011G089900* (a homolog of *A. thaliana CSLA2*), which were enriched in both the ‘cell wall organization’ and ‘external encapsulating structure organization’ GO-BP category (Fig. 4D and E). These findings suggest that GPM1 may enhance graft adhesion by upregulating genes involved in cell wall remodeling and morphogenesis. In contrast, 2,4-D-induced upregulated genes revealed significant enrichment of BPs related to cell proliferation, including ‘cell cycle’, ‘cell division’, and ‘mitotic cell cycle’ (Fig. 4D). Additionally, auxin-responsive genes were strongly induced by 2,4-D but not by GPM1 (Fig. S5), indicating that GPM1 does not activate canonical auxin response during graft tissue adhesion. To directly compare transcriptional responses induced by GPM1 and 2,4-D during graft adhesion, we analyzed treatment-specific and shared DEGs (Fig. S6). Only 17 upregulated genes were common to both treatments, whereas 2,4-D and GPM1 uniquely induced 2387 and 32 genes, respectively. The shared DEGs were primarily associated with cell wall modification and stress responses. In contrast, 2,4-D-specific DEGs were strongly enriched in cell cycle and cell division-related processes, while GPM1-specific DEGs were enriched in secondary metabolism and phenylpropanoid-related pathways. These results indicate that, although GPM1 and 2,4-D share a limited transcriptional response, GPM1 preferentially modulates pathways related to cell wall remodeling, which may facilitate tissue adhesion at the graft interface. Consistent with this interpretation, co-treatment with 2,4-D and GPM1 did not result in a synergistic enhancement of graft adhesion (Fig. S7), suggesting that the two compounds may act through partially overlapping pathways which associated with cell wall remodeling.

### GPM1 promoted scion growth and callus cell expansion in *G. max* stem grafting

**Figure 5:**
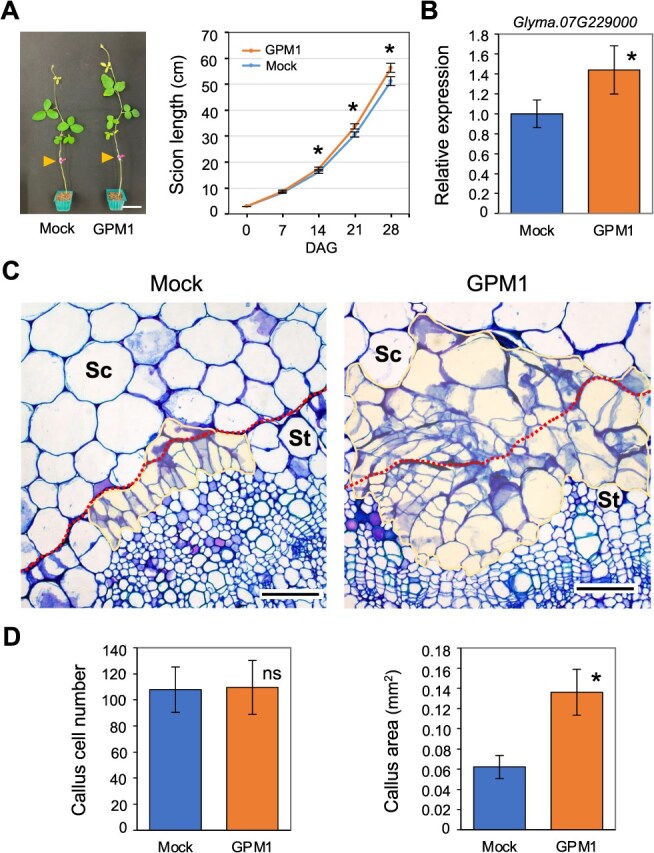
Scion growth and histological characterization of *Glycine max* self-grafting treated with GPM1. (A) Representative image of stem grafts treated with mock (0.1% DMSO) or 10 μM GPM1 at 28 DAG. Arrowheads indicate the graft junction. Scale bar = 5 cm. Scion length was measured at 0, 7, 14, 21, and 28 DAG. Error bars indicate the means ± SE (*n* = 80–88). Asterisks indicate significant differences versus mock (Mann–Whitney *U* test, *P* < 0.05). (B) Relative expression of *Glyma.07G229000* at 1 DAG measured by qRT-PCR, normalized to *EF1α*. Error bars indicate the means ± SE (*n* = 23). Asterisk indicates a significant difference versus mock (Student’s *t*-test, *P* < 0.05). (C) Histological cross sections of graft junctions at 7 DAG (12 μm thickness, stained with toluidine blue). Yellow indicates callus, red dashed lines indicate the graft interface. Sc, scion; St, rootstock. Scale bars = 100 μm. Original images were presented in Fig. S6. (D) Quantification of callus cell number and area in the pith using ImageJ. Error bars indicate the means ± SE (*n* = 7). ns, not significant. Asterisk indicates a significant difference versus mock (Mann–Whitney U test, *P* < 0.05).

To validate the function of GPM1 in enhancing graft tissue adhesion observed in four Fabaceae species within the IVG system, we subsequently evaluated its effect on stem grafting in *G. max*. Ten-day-old *G. max* seedlings were self-grafted and treated with either mock (0.1% DMSO) or 10 μM GPM1 at the graft junction and scion length from the graft junction was measured at 0, 7, 14, 21, and 28 DAG. GPM1-treated scions exhibited significantly increased scion growth compared to the mock-treated scions at 14, 21, and 28 DAG (Fig. 5A), indicating that the graft promoting effect of GPM1 enhanced scion growth in *G. max* stem grafting.

We next confirmed upregulation of expansin genes in the GPM1 treated grafts. Since *Phvul.002G083600* (*PvEXPA5*) gene showed significant upregulation based on -log_10_(padj) values (Fig. 4C, Table S1) in the RNA-seq analysis of *P. coccineus* IVG at 1 DAG, we focused on its close homologs. Phylogenetic analysis showed *Phvul.002G083600* (*PvEXPA5*) and *Phvul.002G170300* (*PvEXPA7*) are two closest homologs and four homologs exist in *G. max* (Fig. S8A and B). In *P. coccineus* IVG, both the closest homologs, *Phvul.002G083600* (*PvEXPA5*) and *Phvul.002G170300* (*PvEXPA7*) were significantly upregulated in GPM1-treated IVG samples compared to the mock (Fig. S8C). In *G. max* stem grafts, *Glyma.07G229000* was significantly upregulated by GPM1 treatment compared to the mock (Fig. 5B), while the other three homologs were not regulated (Fig. S8D).

To further investigate the mechanism behind the graft promotion by GPM1, we conducted histological analyses of the graft junctions. Graft samples were collected at 7 DAG, resin-embedded, and examined. We observed larger callus cells in the pith region at the graft interface of GPM1-treated grafts compared to mock-treated grafts (Fig. 5C, Fig. S9). Quantitative analysis revealed no significant difference in the number of callus cells between the treatments; however, the total callus area was significantly greater in GPM1-treated grafts than in the mock (Fig. 5D), indicating that GPM1 promotes cell expansion in the callus tissue.

## Discussion

### GPM1 promotes graft adhesion across multiple species in Fabaceae and others

Using a modified IVG system, we screened artificially synthesized 3000 compounds and identified GPM1, which significantly enhanced graft tissue adhesion in *P. coccineus*. The combination of qualitative hand tests and quantitative force measurements ensured robust and precise evaluation.

GPM1 promoted graft adhesion in IVG grafts of *P. coccineus*, *V. unguiculata*, *V. angularis*, and *G. max*, although the effect varied among species, with *V. unguiculata* showing the strongest response and *G. max* the weakest, highlighting the species-specific nature of graft compatibility, likely governed by a combination of physiological and molecular factors [46]. Importantly, GPM1 also enhanced adhesion in heterografts, such as *V. unguiculata* scions grafted onto *G. max* rootstocks (Fig. 3), suggesting that its graft-promoting activity extends to interspecific graft combinations. In stem grafting experiments, *V. unguiculata*/*G. max* grafts treated with GPM1 tended to show higher survival rates than mock-treated controls, although this increase did not reach statistical significance. Moreover, beyond legumes, GPM1 also increased graft adhesion in *N. benthamiana* IVG grafts and significantly improved survival rates in *A. thaliana* micrografting (Figs S3 and S4). This property positions GPM1 as a broadly applicable chemical tool for improving graft performance, with the extent of grafting promotion depending on the graft combination.

### Transcriptomic insights into GPM1-induced grafting enhancement

Transcriptome analysis revealed that GPM1 induces a distinct and targeted gene expression pattern compared to 2,4-D. In *P. coccineus* IVG grafts, GPM1 upregulated several genes associated with cell wall remodeling, a key process in graft union formation. Notably, expansin genes (*PvEXPA5*, *PvEXPA22*, *PvEXPA25*) were strongly induced. Expansins are known to loosen the cell wall matrix, thereby facilitating cell expansion and tissue adhesion during graft healing [26, 47]. In addition to expansins, GPM1 also induced *Phvul.011G089900* (a homolog of *A. thaliana CSLA2*), involved in hemicellulose biosynthesis, *Phvul.011G055900* (a homolog of *A. thaliana BGLU15*), a β-glucosidase implicated in cell wall remodeling, *Phvul.001G209100* (a homolog of *A. thaliana PME41*), a pectin methylesterase inhibitor that modulates cell wall plasticity, and *Phvul.007G228000* (a homolog of *A. thaliana HPT1*), a hydroxyproline-rich glycoprotein linked to cell signaling and adhesion. Together, the induction of these genes suggests that GPM1 may enhance graft adhesion by activating coordinated cell wall modification at the graft interface. The 2,4-D treatment primarily upregulated genes involved in the cell cycle and cell division, but also significantly induced a range of cell wall-related genes, including members of the *EXPA*, *EXPB*, *EXPR*, *XTH*, *PMEPCRA/B*, *PME*, *GH9B3*, and *CSLA* families. These results are consistent with previous studies showing that auxin modulates cell wall structure [47, 48]. Together, these findings suggest that auxin may enhance graft adhesion not only by stimulating cell proliferation but also by altering the mechanical properties of the cell wall at the graft interface through coordinated gene activation.

Co-treatment with 2,4-D and GPM1 did not result in a synergistic enhancement of graft adhesion (Fig. S7), suggesting that the two compounds may act through partially overlapping pathways, particularly those involved in cell wall remodeling. This interpretation is supported by RNA-seq data, which showed that both treatments upregulated *PvEXPA5*, *PvEXPA22*, and *PvEXPA25*. However, GPM1 induced a stronger and more specific upregulation of *PvEXPA5*, whereas 2,4-D preferentially upregulated *PvEXPA22* and *PvEXPA25* (Fig. 4E). In *A. thaliana*, treatment with 0.1 μM or higher concentrations of 2,4-D inhibited primary root elongation, a typical auxin response, whereas GPM1 at the tested concentrations did not affect root growth (Fig. S2A and B). Furthermore, *DR5::GFP* signal expansion did not observed in GPM1 treated *A. thaliana* roots (Fig. S2C). Together, these results suggest that GPM1 does not elicit a canonical auxin response, while partially overlapping with auxin in the regulation of cell wall-associated genes during graft healing.

### GPM1 enhances scion growth and callus development in *G. max*

The enhancement of scion growth in grafted *G. max* following the application of GPM1 suggests good tissue reunion and functional integration between graft partners. Notably, GPM1 did not significantly affect graft survival rate compared to the mock at 28 DAG. The survival rate was 90% in the mock-treated grafts and 92.5% in the GPM1-treated grafts. Statistical analysis indicated no significant difference between the two treatments, likely because *G. max* homografts already exhibit a high success rate (>90%) even without treatment (Fig. S10). Furthermore, quantitative analysis of yield-related traits under greenhouse conditions at 100 DAG showed that pod number per graft did not differ significantly between mock and GPM1 treatment, whereas pod weight, seed number, and seed weight per graft showed slightly higher median values in GPM1-treated plants. However, these differences were not statistically significant compared with the mock control (Fig. S11). These results indicate that GPM1 primarily promotes scion stem growth without altering graft survival or yield. Histological analysis at 7 DAG revealed substantial enlargement of callus cells at the graft interface in GPM1-treated plants, particularly within the pith region (Fig. 5C and D). This anatomical change aligns with transcriptomic data showing upregulation of genes related to cell wall loosening and expansion (Fig. 4E), including members of the *EXPA* gene family and other remodeling enzymes. Enlarged callus cells likely contribute to enhanced mechanical continuity and cell–cell adhesion at the graft site, which are critical for establishing a stable graft union. Increased cell wall extensibility may enable callus cells to expand more rapidly and effectively occupy gaps at the graft interface, thereby strengthening tissue integration during graft healing. The conserved activation of expansins in response to GPM1 in *G. max* was observed in the *Glyma.07G229000* which showed significantly increased expression in GPM1-treated samples compared to the mock following qRT-PCR at the graft junction (Fig. 5D).

### Perception and signaling of GPM1

The molecular mechanism underlying plant perception of GPM1 remains unknown. Based on its chemical properties, GPM1 is predicted to be membrane-permeable, as indicated by its cLogP value (4.47), suggesting that it may access intracellular or membrane-associated targets. However, GPM1 does not induce canonical auxin responses, including DR5 reporter activation or typical auxin-related root phenotypes, indicating that it is unlikely to act through the auxin signaling pathway. Because cell wall loosening is often associated with pH-dependent mechanisms such as acid growth, we examined whether GPM1 alters the pH of the culture medium. Measurement of the medium pH showed values of 5.61 in the original medium, 5.57 in the mock control, and 5.62 in the 10 μM GPM1 treatment, which were comparable to those observed in the 0.5 μM 2,4-D treatment (pH 5.58). These results indicate that GPM1 treatment does not cause a detectable change in medium pH (Table S6). Although localized or transient apoplastic acidification in plants cannot be excluded, these observations suggest that GPM1 does not primarily promote graft adhesion through auxin-mediated acid growth, but instead acts via an alternative pathway that converges on cell wall remodeling. Further studies, including direct analysis of apoplastic pH dynamics and identification of GPM1-interacting proteins, will be helpful to elucidate the precise perception and signaling mechanisms of this graft-promoting molecule.

## Materials and methods

### Plant materials and growth conditions

Seeds of *P. coccineus*, *V. unguiculata*, *V. angularis*, and *G. max* were sown in pots (6.5 × 6.5 × 5.0 cm; Canelon Chemical) filled with a 1:1 mixture of Hanachan culture soil (Hanagokoro) and vermiculite (Nittai). Pots were placed on trays and covered with plastic wrap to maintain humidity during germination, which was removed immediately after emergence. Plants were grown at 22°C under continuous light, irrigated three times per week, and supplied with a water-soluble fertilizer (1 g·l^−1^ Hyponex N15P30K1; Hyponex Japan Ltd) during routine watering. *Nicotiana benthamiana* seeds were surface-sterilized with 5% (w/v) sodium hypochlorite for 5 min, rinsed six times with sterile water, and stratified at 4°C in the dark for 3 days. Seeds were sown on half-strength Murashige and Skoog (1/2 MS) medium containing 0.5% sucrose and 1% agar (pH 5.7). After 7 days, seedlings were transferred to soil and grown at 27°C under continuous light (100 μmol m^−2^·s^−1^) with 70% relative humidity, watered three times per week, and fertilized with 1 g·l^−1^ Hyponex.

### *In vitro* grafting

IVG was conducted using sterile 8-well rectangular dishes with lids (127.8 × 85.5 mm; Thermo Fisher). Each well contained 4 ml of autoclaved medium comprising 1/2 MS basal salts (pH 5.7), 1% (w/v) agar, and 300 ng/ml cefotaxime sodium salt (CSS; Tokyo Chemical Industry). Test compounds (10 μM) and control treatments [0.1% (v/v) dimethyl sulfoxide (DMSO), or 0.5 μM 2,4-D] were incorporated into the medium prior to solidification. The pH of the culture medium was measured using a pH meter (F-51; Horiba) equipped with a glass electrode (JF-15; Horiba). We used 2,4-D as a positive control because it is a stable and potent synthetic auxin known to promote callus formation and tissue regeneration [49]. In contrast, indole-3-acetic acid (IAA) is chemically unstable, rapidly photodegraded in culture media, and quickly metabolized in planta, making it less suitable for reproducible *in vitro* assays [50]. Moreover, 2,4-D has been reported to enhance graft adhesion in an IVG system, supporting its use as an appropriate positive control [32]. For combined treatments, 0.5 μM 2,4-D and 10 μM GPM1 were co-applied (Fig. S7). Sterile filter paper (2.5 × 3.5 cm) and IVG silicone sheets were placed in each well. Plant materials included epicotyls from 10-day-old *P. coccineus*, hypocotyls from 10-day-old *V. unguiculata*, 20-day-old *V. angularis*, 10-day-old *G. max* and 4-week-old *N. benthamiana* stems. All tissues were surface-sterilized with 70% ethanol for 30 s and rinsed three times with sterile distilled water. Stem segments were cut into 3 mm lengths, then bisected to yield two 1.5-mm slices. These were reassembled in original polarity and inserted into the IVG sheet cutouts using sterile tweezers. Plates were sealed with surgical tape and incubated at 27°C under continuous light. Adhesive force was measured using a digital force gauge.

### Chemical screening

A total of 3000 compounds from the ITbM Library were screened. Compounds were dissolved in DMSO (10 mM stock) and incorporated into 1/2 MS medium (pH 5.7) supplemented with 1% agar and 300 ng/ml CSS. Final concentrations were 10 μM for test compounds, 0.1% (v/v) DMSO for the negative control, and 0.5 μM 2,4-D for the positive control. Epicotyls from 10-day-old *P. coccineus* were used as graft material. In the first and second screening rounds, 3 IVG grafts were prepared per treatment. Grafts were manually evaluated at 5 DAG by gently pulling the two tissue slices apart with tweezers. Compounds for which two or more grafts exhibited detectable adhesion were advanced to the second round, which used the same criteria. In the third and fourth rounds, 24 IVG grafts were prepared per treatment. Adhesive force was quantified at 5 DAG using a digital force gauge. Statistical significance compared to DMSO-treated controls was assessed using the Mann–Whitney U test. Compounds showing a significant increase in adhesive force (*P* < 0.05) in both rounds were defined as hit compounds. Chemical characterization was performed using ChemDraw (ChemDraw Prime, Revvity Signals Software, Inc).

### Transcriptome analysis

Epicotyls from 10-day-old *Phaseolus coccineus* seedlings were used for IVG and treated with mock (0.1% DMSO), 0.5 μM 2,4-D, or 10 μM GPM1. IVG procedures and compound application were performed as described above. Three biological replicates were analyzed, each consisting of pooled tissues from 24 IVG grafts. Grafts were harvested at 1 day after grafting. Total RNA was extracted using the RNeasy Mini Kit (Qiagen) according to the manufacturer's instructions. RNA sequencing was conducted at the Nagoya University Gene Experimental Facility. Raw reads were quality-checked and trimmed using fastp, then mapped to the *P. vulgaris* reference genome (Pvulgaris_442_v2.1) with HISAT2. SAM files were converted to BAM format using SAMtools v1.4.1, and transcript assembly was performed with StringTie. Gene expression levels were quantified as transcripts per million (TPM). DEGs were identified using DESeq2. Principal component analysis, DEG analysis, and volcano plots were generated using DEBrowser v1.26.3. GO enrichment analysis was performed using ShinyGO v0.82. Hierarchical clustering of auxin-responsive genes and the 17 DEGs commonly induced by 2,4-D and GPM1 was conducted in R and Venn diagrams were generated using Venny 2.1.0.

### Stem grafting

Stem grafting of 10-day-old *G. max* was performed using the splice graft method. For each plant, one cotyledon and one true leaf at the same position were removed to reduce water loss. The plants were cut diagonally 3 cm below the apex, and the graft junction was trimmed to 1.5 cm. Scion and rootstock were aligned at their cut surfaces, wrapped with gauze, and secured with a grafting clip. For treatments, 200 μl of 0.1% DMSO (mock) or 10 μM GPM1 was applied to the gauze with a pipette immediately after grafting, followed by an additional 100 μl at 3 DAG. To prevent drying, the plants were misted twice with tap water and covered with a plastic bag (Unipak F-4; Seisannipponsha Ltd). Grafted plants were incubated at 27°C. At 6 DAG, the cover was removed, and at 7 DAG, plants were moved to continuous light at 22°C. Plants were watered three times per week, using 1 g/l Professional Hyponex (N15P30K1). Each treatment included 80–88 grafted plants. Scion growth was measured at 0, 7, 14, 21, and 28 DAG using a ruler, from the grafting site to the scion’s terminal bud.

### Quantitative RT-PCR

The 10-day-old *G. max* was used to perform stem grafting and the grafts were treated with mock (0.1% DMSO) and 10 μM GPM1, separately. Quantitative reverse transcription polymerase chain reaction (qRT-PCR) was performed using tissue pooled from 6 stem grafts per treatment at 1 DAG for *Glyma.01G050100*, *Glyma.02G109100*, and *Glyma.20G033900*, and from 23 stem grafts per treatment for *Glyma.07G229000*. The methods of stem grafting and treatment addition were conducted in the same way as mentioned above. Total RNA was extracted from grafting sites using the TRIzol Reagent protocol (15 596 026; Thermo Fisher). First-strand cDNA synthesis was carried out using the SuperScript III First-Strand Synthesis Super Mix (18080-400; Thermo Fisher) according to the manufacturer's instructions. qRT-PCR was performed using the KAPA SYBR FAST qPCR Master Mix (2×) Kit (ROX Low, 10 μl reaction) in 96-well optical plates on a QuantStudio 3 Real-Time PCR System (Thermo Fisher) under standard thermocycler conditions. Relative expression values are given as the means of three technical replicates, which is relative to the control using *EF1α* (*Glyma.19G052400*) as the housekeeping gene. Data were analyzed using Student’s *t*-test (*P* < 0.05). The primers used for PCR amplification are listed in the Table S5.

### Microscopy and callus measurement

Stem grafting was performed on 10-day-old *G. max* seedlings using the method described above. Grafts were treated with either mock (0.1% DMSO) or 10 μM GPM1. At 7 DAG, a 0.5 cm segment of the graft junction from each of seven grafts per treatment, collected at the same position, was used for histological analysis. Samples were fixed and embedded using the Technovit 7100 resin kit (Kulzer), sectioned with a rotary microtome (RX-860, Yamato Kohki), and stained with 0.02% toluidine blue for 3 to 5 min. Sections were rinsed with sterile water, air-dried, and imaged under a light microscope (BX53, Olympus) equipped with a digital camera (DP73, Olympus). For callus evaluation, one cross-section per 0.5 cm segment at the same position was analyzed in the pith region around the graft interface. Callus cell number and area were quantified using ImageJ. Representative cross-sectional images of *G. max* graft junctions treated with mock or GPM1 at 7 DAG are shown in Fig. S9.

## Supplementary Material

Web_Material_uhag095

## Data Availability

RNA-Seq data are available from the DNA Data Bank of Japan (DDBJ; http://www.ddbj.nig.ac.jp/) under the accession number PRJDB37567. All other data supporting this study are available in the main text and supplementary materials.
