# Supplementary material for: Chemical screening in Fabaceae identified GPM1 as a novel compound enhancing early graft adhesion
Source: Hortic Res. 2026 Mar 13;13(7):uhag095. doi: 10.1093/hr/uhag095 (PMC13271802; doi:10.1093/hr/uhag095)
Supplement: Web_Material_uhag095 [file web_material_uhag095.zip › Luo_et_al_Supplemental Material.pdf]

Supplemental Material

Chemical Screening in Fabaceae Identified GPM1 as a Novel Compound Enhancing Early Graft Adhesion

Qianqian Luo, Xueyao Shu, Ayato Sato, Yaichi Kawakatsu, Kentaro Okada, Frank Opoku-Agyemang, Ken-ichi Kurotani, Michitaka Notaguchi

Supplemental Materials and Methods

Supplemental Figure S1. Time-course analysis of *in vitro* grafting adhesive force in *Phaseolus coccineus.*

Supplemental Figure S2. Root phenotype and *DR5::GFP* reporter analysis in *Arabidopsis thaliana* treated with 2,4-D and GPM1.

Supplemental Figure S3. GPM1 enhanced graft adhesion in *Nicotiana benthamiana* at 7 days after grafting.

Supplemental Figure S4. GPM1 improved survival rate in *Arabidopsis thaliana* at 14 days after grafting.

Supplemental Figure S5. Expression profiles of auxin-responsive genes.

Supplemental Figure S6. Comparison of transcriptomic responses to 2,4-D and GPM1 treatment in *Phaseolus coccineus*.

Supplemental Figure S7. Co-treatment effects of 2,4-D and GPM1 on *in vitro* grafting adhesion.

Supplemental Figure S8. Phylogenetic analysis and expression of expansin genes.

Supplemental Figure S9. Histology of *Glycine max* stem grafts treated with mock and GPM1.

Supplemental Figure S10. Survival rate of *Glycine max* self-grafting treated with GPM1.

Supplemental Figure S11. Yield analysis of *Glycine max* self-grafting treated with GPM1.

Supplemental Materials and Methods

Root phenotype analysis and *DR5::GFP* signal observation in *Arabidopsis thaliana*

The *DR5::GFP* seeds, N9361, were obtained from the Nottingham Arabidopsis Stock Centre (NASC). Seeds were incubated at 4°C in the dark for 3 days and germinated on 1/2 MS medium supplemented with 1% (w/v) sucrose at 22°C under continuous light for 3 days. For root growth assays, 3-day-old seedlings were transferred to 1/2 MS medium containing mock (0.1% DMSO), 2,4-D, or GPM1 at 0.01, 0.1, 1, or 10 μM and grown for an additional 8 days under continuous light. Root length was measured using ImageJ ($n = 18$). For auxin-response analysis, *A. thaliana DR5::GFP* seedlings were grown for 3 days on 1/2 MS medium with 1% sucrose and then treated with mock (0.1% DMSO), 2,4-D, or GPM1 at 0.1, 1, or 10 μM for 24 h at 22°C under continuous light. Prior to imaging, roots were stained with propidium iodide (PI; 20 μg mL^{-1}). Fluorescence was visualized using a confocal laser microscope (FV3000; Olympus, Tokyo, Japan). For GFP detection, samples were excited at 488 nm with emission collected between 500 and 540 nm. PI fluorescence was excited at 561 nm and detected in the 570–620 nm range. A total of six seedlings were analyzed per treatment.

Arabidopsis thaliana micrografting

A. thaliana micrografting was performed as described by Tsutsui et al. (2020). Briefly, Lanolin (1 mL; FUJIFILM Wako Chemicals) was melted at 60°C and mixed with 200 μL of mock solution (0.1% DMSO), 0.1 μM 2,4-D, or 10 μM GPM1. Approximately 5 μL of the lanolin mixture was applied to the scion region immediately before grafting. Grafting success was evaluated at 14 days after grafting (DAG). Grafts were considered successful when scion leaves turned green, new leaves emerged, and root elongation was observed; grafts showing dark-green scion leaves without growth were scored as failed.

Phylogenetic analysis

Phylogenetic analysis was conducted to investigate expansin homologs in *G. max* based on the phylogenetic relationship with expansin genes from *P. vulgaris* and *A. thaliana*. A total of 35 expansin protein sequences from *A. thaliana* were retrieved from the Araport11 database (Araport11_pep_20240409), 42 sequences from *P. vulgaris* from Phytozome v13 (Pvulgaris_442_v2.1.protein) and 89 sequences from *G. max* from Phytozome v13 (Gmax_880_Wm82.a6.v1.protein). Multiple sequence alignment was performed using the ClustalW algorithm. A phylogenetic tree was then constructed using the Neighbor-Joining method implemented in MEGAX, applying the p-distance substitution model with uniform rates. Gaps and missing data were handled using pairwise deletion. The reliability of the tree topology was assessed by 1,000 bootstrap replicates. The *PvEXPA5*-related clade was extracted from the full expansin phylogenetic tree.

Yield analysis of *Glycine max* self-grafting

Yield analysis of *G. max* self-grafting was performed under greenhouse conditions to evaluate the long-term effects of GPM1 treatment. Splice grafting was conducted using 10-day-old *G. max* seedlings. Grafted plants were initially maintained in a growth chamber under continuous light conditions at 27°C from 0 to 7 DAG, followed by continuous light conditions at 22°C from 7 to 14 DAG in a growth room. Thereafter, plants were transferred to greenhouse and cultivated under a photosynthetic photon flux density of approximately 1,000 μmol m^{-2} s^{-1} at 25°C until harvest.

Treatments included mock control (0.1% DMSO) and 10 μM GPM1. A total of 28 grafted plants were used per treatment. At 100 DAG, yield-related traits were evaluated, including pod number, pod weight, seed number, and seed weight per graft. Statistical comparisons between treatments were conducted using the Mann-Whitney U test.

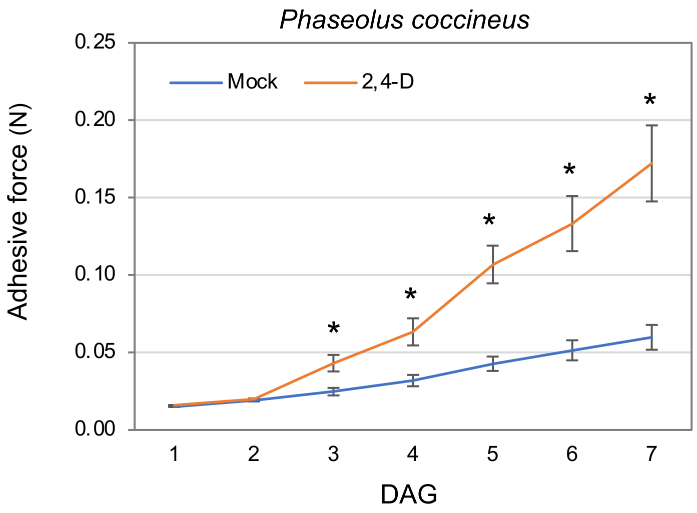

Supplemental Figure S1. Time-course analysis of *in vitro* grafting adhesive force in *Phaseolus coccineus*. Adhesive force of IVG grafts in the epicotyl of 10-day-old *P. coccineus*, measured at 1, 2, 3, 4, 5, 6, and 7 days after grafting (DAG). Treatments included mock (0.1% DMSO) and 0.5 μM 2,4-D. Asterisks indicate statistically significant differences compared to the mock (Mann-Whitney U test, $P < 0.05$). Error bars indicate the means ± SE (n = 40–47).

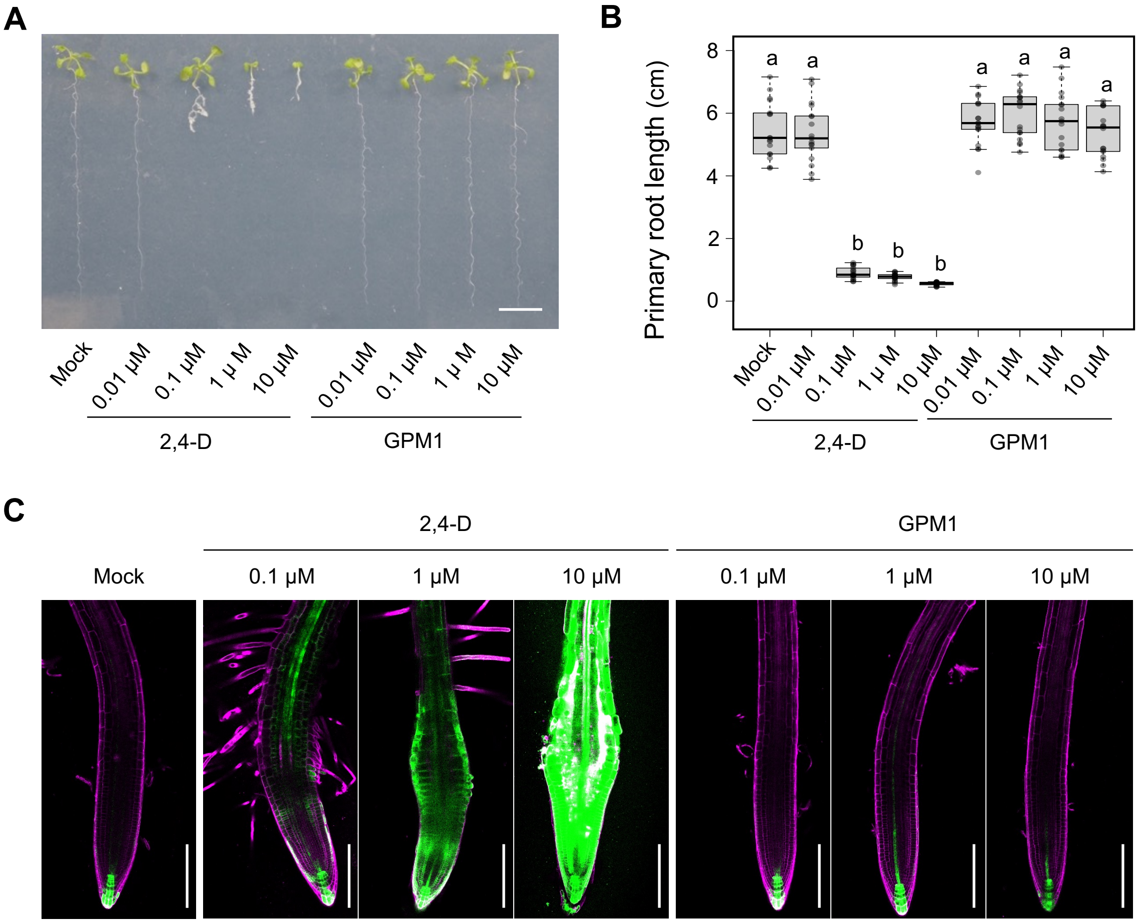

Supplemental Figure S2. Root phenotype and *DR5::GFP* reporter analysis in *Arabidopsis thaliana* treated with 2,4-D and GPM1. A. Representative image of 11-day-old *A. thaliana* seedlings grown on media supplemented with mock (0.1% DMSO), 2,4-D at 0.01, 0.1, 1, and 10 µM, and GPM1 at the same concentrations. Scale bar = 1 cm. **B.** Quantification of primary root length. $n = 18$. Data were analyzed using the Tukey's HSD test for multiple comparisons ($P < 0.05$). Different letters denote statistically significant differences. **C.** Visualization of auxin-responsive regions using *DR5::GFP* to assess auxin-like activity of GPM1. Three-day-old *A. thaliana* seedlings were treated with 2,4-D or GPM1 at concentrations of 0.1, 1, and 10 µM for 24 h. Green indicates GFP fluorescence, and magenta indicates propidium iodide staining (20 µg mL^{-1}). Scale bar = 200 µm.

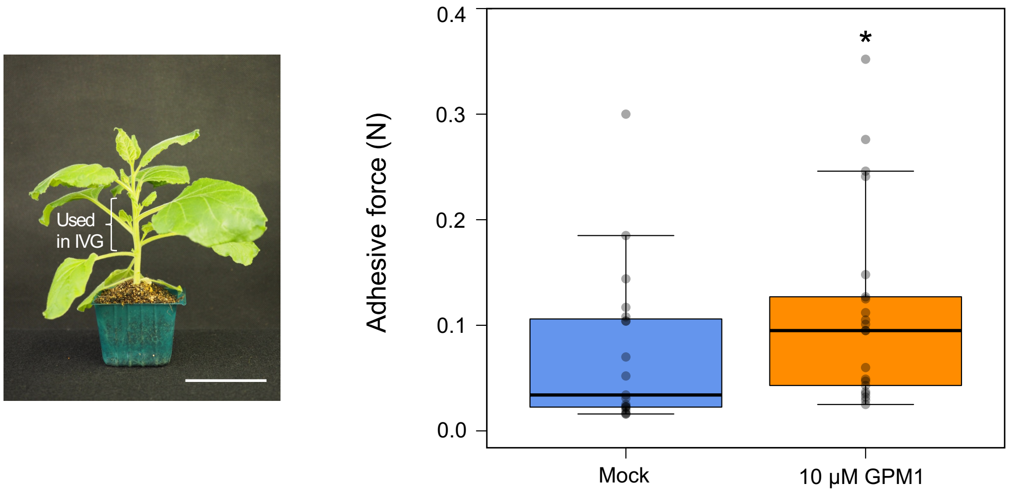

Supplemental Figure S3. GPM1 enhanced graft adhesion in *Nicotiana benthamiana* at 7 days after grafting. *In vitro* grafting (IVG) was performed using stems of 4-week-old *N. benthamiana* plants (n = 19–21). A representative *N. benthamiana* plant is shown. Scale bar = 5 cm. Treatments included mock (0.1% DMSO) and 10 µM GPM1. Asterisk indicates a significant difference compared with the mock (Mann-Whitney U test, $P < 0.05$).

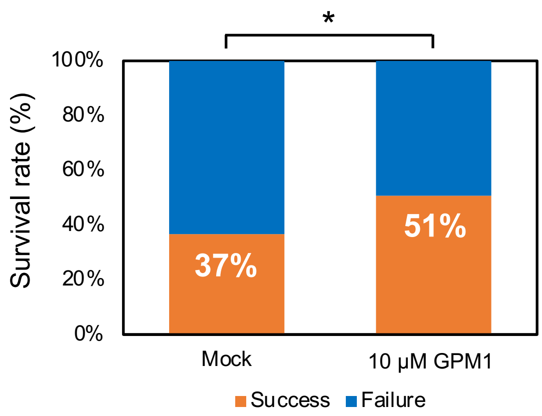

Supplemental Figure S4. GPM1 improved survival rate in *Arabidopsis thaliana* micrografts at 14 days after grafting. Hypocotyl micrografting was performed using 4-day-old *A. thaliana* seedlings (n = 118–120). Treatments included mock (0.1% DMSO) and 10 μM GPM1. Asterisk indicates a significant difference compared with the mock (Chi-square test, $P < 0.05$).

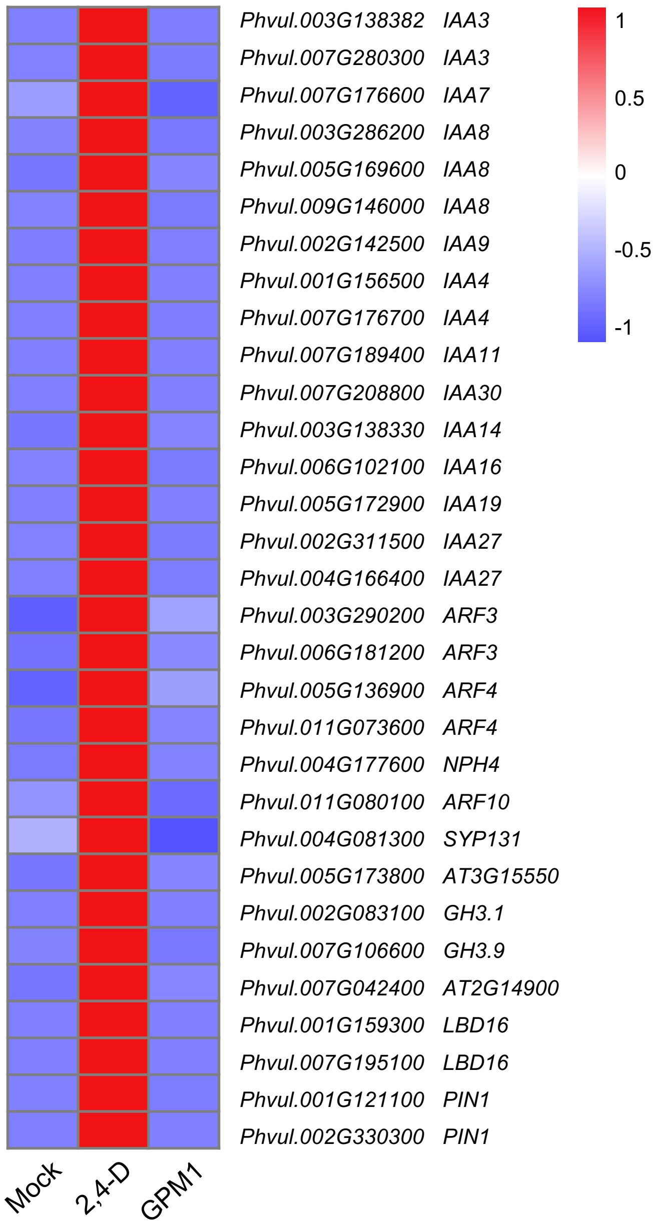

Supplemental Figure S5. Expression profiles of auxin-responsive genes. Heatmap showing expression of auxin-responsive genes in *Phaseolus coccineus* IVG grafts at 1 day after grafting treated by mock (0.1% DMSO), 0.5 µM 2,4-D or 10 µM GPM1.

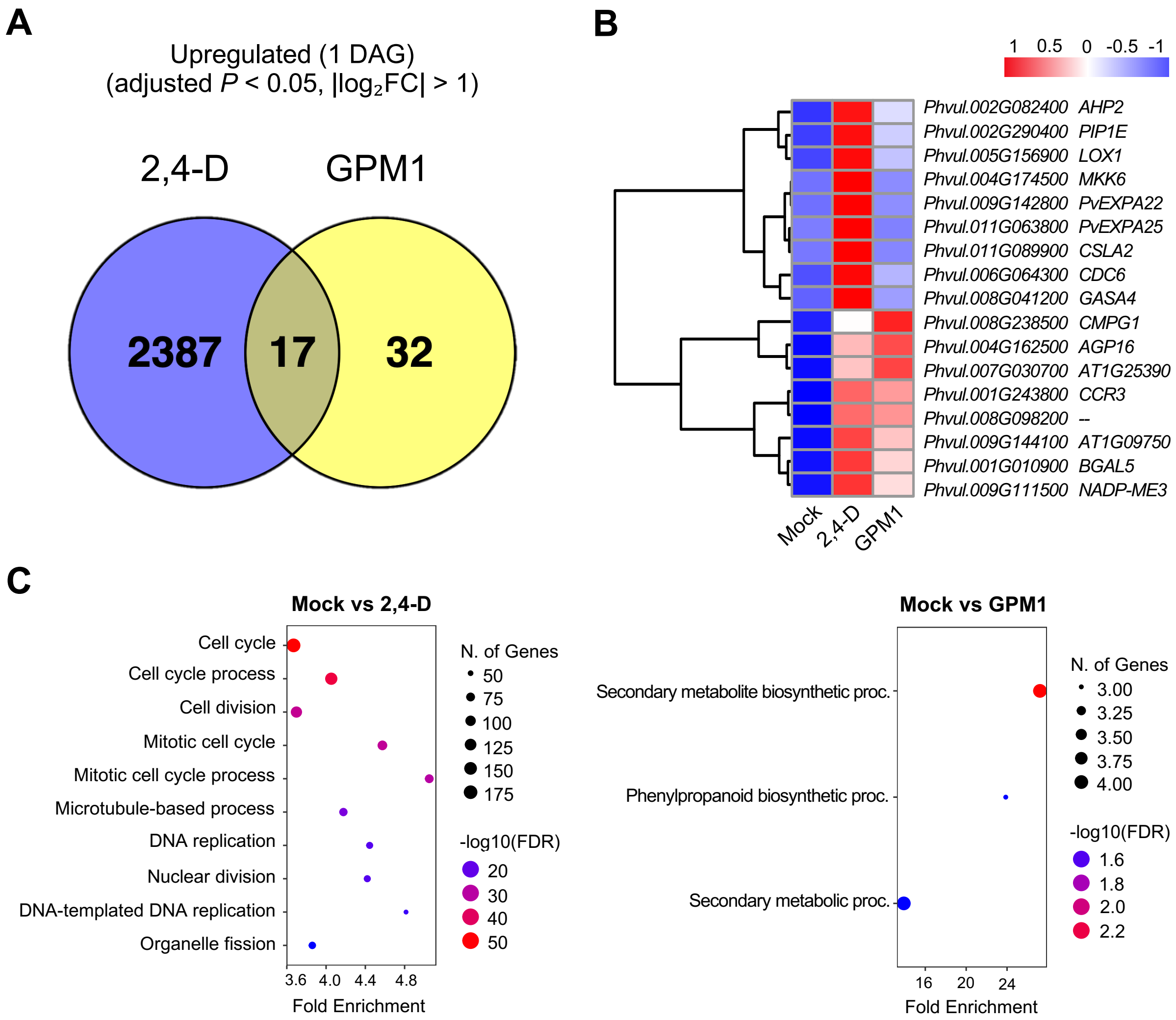

Supplemental Figure S6. Comparison of transcriptomic responses to 2,4-D and GPM1 treatment in *Phaseolus coccineus*. A. Venn diagrams showing upregulated differentially expressed genes (DEGs; adjusted $P < 0.05$, $|\log_2$ fold change$| > 1$) after 1 day of treatment with 0.5 µM 2,4-D or 10 µM GPM1. **B.** Heatmap showing the expression patterns of the 17 DEGs commonly induced by both 2,4-D and GPM1 in *P. coccineus in vitro* grafting samples at 1 day after grafting (DAG) treated with mock (0.1% DMSO), 0.5 µM 2,4-D, or 10 µM GPM1. Genes are labeled using *A. thaliana* annotation names. *P. vulgaris* gene nomenclature follows the Expansin Gene Family Database. **C.** Gene Ontology enrichment analysis of 2,387 upregulated DEGs uniquely induced by 2,4-D and 32 upregulated DEGs uniquely induced by GPM1. The top 10 enriched Biological Process terms are shown for each treatment.

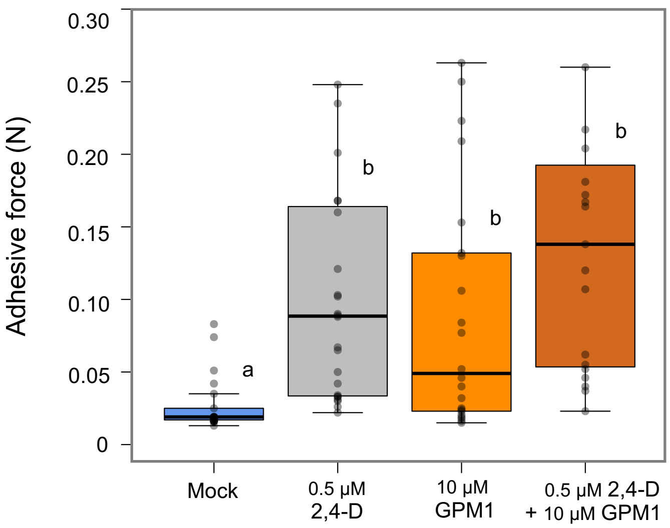

Supplemental Figure S7. Co-treatment effects of 2,4-D and GPM1 on *in vitro* grafting adhesion. Adhesive force of *Phaseolus coccineus* IVG grafts measured at 5 days after grafting. Treatments included mock (0.1% DMSO), 0.5 μM 2,4-D, 10 μM GPM1 and a combination treatment of 0.5 μM 2,4-D and 10 μM GPM1. n = 14–25. Different letters indicate statistically significant differences between treatments determined by the Steel-Dwass' test ($P < 0.05$).

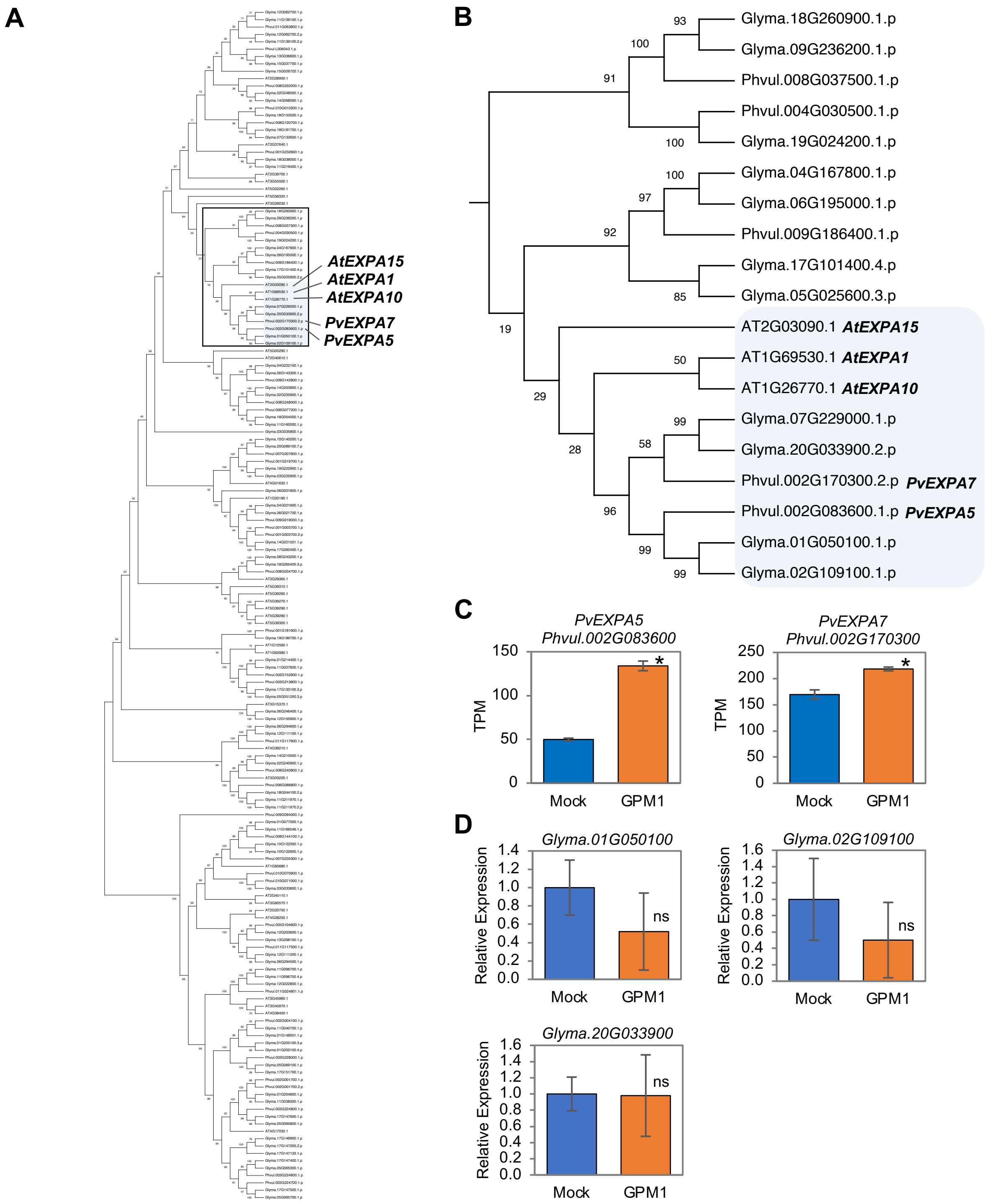

Supplemental Figure S8. Phylogenetic analysis and expression of expansin genes. A. Phylogenetic relationships of expansin homologs in *Glycine max*, *Phaseolus vulgaris*, and *Arabidopsis thaliana*. The *PvEXPA5* (*Phvul.002G083600*) -related clade is indicated. **B.** Enlarged view of the *PvEXPA5*-related clade. Homologs of *PvEXPA5* are highlighted in blue. *P. vulgaris* gene nomenclature follows the Expansin Gene Family Database. **C.** GPM1 significantly upregulated *PvEXPA5* and *PvEXPA7* (*Phvul.002G170300*) at 1 day after grafting in *P. coccineus in vitro* grafts. Asterisks indicate significant differences ($P < 0.05$, Student's t-test). **D.** Relative expression of the corresponding *G. max* homologs at 1 day after grafting in stem grafts, determined by qRT-PCR. Expression levels were normalized to *EF1α*. Values represent means ± SE ($n = 6$). ns, not significant ($P > 0.05$, Student's t-test).

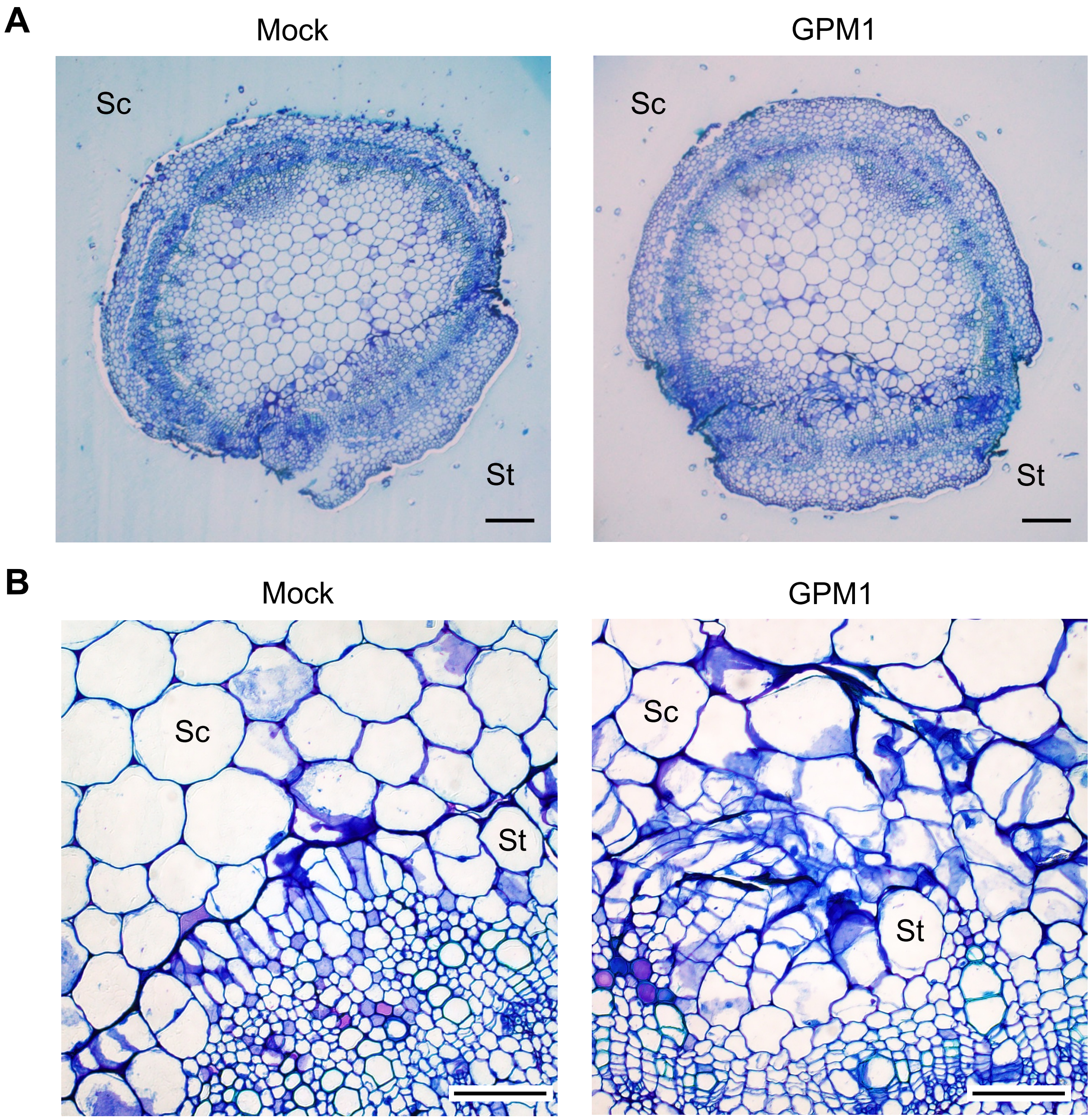

Supplemental Figure S9. Histology of *Glycine max* stem grafts treated with mock and GPM1. Representative cross-sections (12 μm thickness, stained with toluidine blue) of stem graft junctions treated with mock (0.1% DMSO) or 10 μM GPM1 at 7 days after grafting. **A.** Images taken at 4× magnification. Sc, scion; St, rootstock. Scale bar = 200 μm. **B.** Higher-magnification view (20×) of the same sections around the pith region at the graft junction. Scale bar = 100 μm.

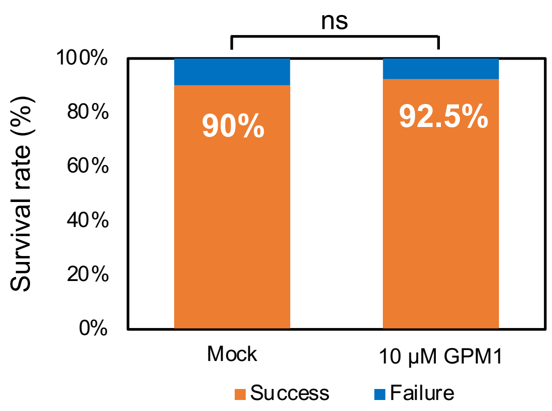

Supplemental Figure S10. Survival rate of *Glycine max* self-grafting treated with GPM1. Survival was assessed at 28 days after grafting (DAG). $n = 40$. ns, not significant (Chi-square test, $P > 0.05$).

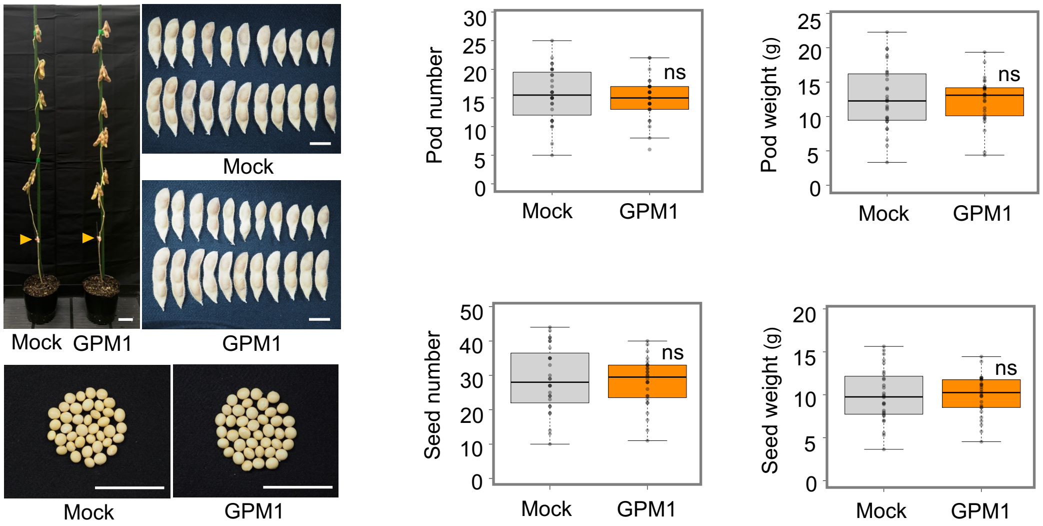

Supplemental Figure S11. Yield analysis of *Glycine max* self-grafting treated with GPM1. Stem grafting was performed using 10-day-old *G. max* seedlings (n = 28). Representative images of grafted plants, pods per graft, and seeds per graft are shown. Arrowheads indicate the graft junction. Scale bars = 5 cm. Quantitative analysis of yield-related traits, including pod number, pod weight, seed number, and seed weight per graft at 100 days after grafting. Treatments included mock (0.1% DMSO) and 10 µM GPM1. ns, not significant compared with mock (Mann-Whitney U test, $P > 0.05$).
